# Thyroglobulin/thyrotropin ratio for predicting long-term response in differentiated thyroid carcinoma: a retrospective study

**DOI:** 10.20945/2359-3997000000387

**Published:** 2021-07-16

**Authors:** Adriano Francisco De Marchi, Ana Bárbara Trizzotti de Macedo, Carlos Segundo Paiva Soares, Fernanda Bolfi, Mariana Riello Gomes Iessi, Cristiano Claudino de Oliveira, Katia Hiromoto Koga, Sonia Marta Moriguchi, José Vicente Tagliarini, Gláucia Maria Ferreira da Silva Mazeto

**Affiliations:** 1 Universidade Estadual Paulista Faculdade de Medicina de Botucatu Departamento de Clínica Médica Botucatu SP Brasil Departamento de Clínica Médica, Faculdade de Medicina de Botucatu, Universidade Estadual Paulista (Unesp), Botucatu, SP, Brasil; 2 Universidade Estadual Paulista Faculdade de Medicina de Botucatu Departamento de Oftalmologia, Otorrinolaringologia e Cirurgia de Cabeça e Pescoço Botucatu SP Brasil Departamento de Oftalmologia, Otorrinolaringologia e Cirurgia de Cabeça e Pescoço, Faculdade de Medicina de Botucatu, Universidade Estadual Paulista (Unesp), Botucatu, SP, Brasil; 3 Universidade Estadual Paulista Faculdade de Medicina de Botucatu Departamento de Patologia Botucatu SP Brasil Departamento de Patologia, Faculdade de Medicina de Botucatu, Universidade Estadual Paulista (Unesp), Botucatu, SP, Brasil; 4 Universidade Estadual Paulista Faculdade de Medicina de Botucatu Departamento de Medicina Nuclear Botucatu SP Brasil Departamento de Medicina Nuclear, Faculdade de Medicina de Botucatu, Universidade Estadual Paulista (Unesp), Botucatu, SP, Brasil

**Keywords:** Thyroid neoplasms, thyroglobulin, prognosis

## Abstract

**Objective:**

Thyrotropin-stimulated thyroglobulin (STg) after total thyroidectomy is a prognosis marker for differentiated thyroid carcinoma (DTC). As Tg level is influenced by thyrotropin (TSH), perhaps the STg/TSH ratio is also a prognosis marker for these tumours. We aimed to compare STg/TSH ratio and first STg level in differentiated thyroid carcinoma patients for their ability to predict the long-term response to initial treatment.

**Subjects and methods:**

This retrospective study evaluated data from 181 DTC patients for first (1^st^) STg and STg/TSH ratio, at 1-3 months post-total thyroidectomy and before iodine-131 therapy, according to response to initial therapy [Excellent/Indeterminate or Incomplete (Biochemical/Structural)] observed at final evaluation, and with the survival time with excellent/indeterminate response.

**Results:**

Cases with incomplete response presented higher STg level [225.13 ± 585.26 ng/mL versus (vs) 20.4 ± 192.9 ng/mL; p < 0.001] and STg/TSH ratio (3.01 ± 7.8 vs 0.27 ± 2.58; p < 0.001). Cutoffs of 5 ng/mL for STg and 0.085 for STg/TSH displayed sensitivities of 76.7% and 76.9%, and specificities of 79.2% and 82.6%, respectively, in predicting response to therapy. Values below these cutoffs were associated with longer survival time in excellent/indeterminate response (140.4 vs 15.9 and 144.6 vs 15.9 months, respectively).

**Conclusion:**

STg/TSH ratio has a similar performance to the 1^st^ STg in predicting long-term response to initial therapy.

## INTRODUCTION

Differentiated thyroid carcinomas (DTC) make up almost all malignant thyroid neoplasias and have been increasing in incidence ( 1 ). The most frequent DTC's are papillary carcinomas (PC) accounting for more than 80% of cases with follicular carcinomas (FC) corresponding to between 10 and 13% ( 2 , 3 ).

DTC's have low lethality rates and slow indolent growth. Although 10 year survival rates are around 80%-95%, some cases have more aggressive behaviour and recurrence levels up to 35% indicating a cautious approach and long-term clinical follow-up ( 4 – 8 ). For a long time the standard treatment for these tumours was total thyroidectomy (TT) followed by iodine-131 (^131^I) therapy and thyrotropin (TSH) suppression with levothyroxine ( 2 ). More recently, more conservative surgical therapies have been proposed along with less rigorous follow-up strategies depending on tumour presentation and behaviour, and case evolution expectations ( 2 ).

Thus, efforts have been made to detect early markers of patient evolution in order to better control the need for additional treatment to thyroidectomy and more rigorous long-term follow-up. Several individual factors have been reported as prognosis indicators; these include patient age and lymph node involvement ( 5 ). Additionally, staging systems applied after initial treatment have been used for this purpose, the most common being from the American Joint Committee on Cancer (AJCC/TNM) ( 2 ). As this system evaluates the risk of death from neoplasia, and as mentioned above, DTC is characterised by its low mortality rates, the American Thyroid Association (ATA) proposed a recurrence risk stratification system, classifying cases as low, intermediate or high risk ( 2 ). Although this system is very useful, it does not evaluate long-term case evolution according to treatment given. Thus, the latest ATA guidelines suggest a new more dynamic classification system based on therapeutic response to initial treatment ( 2 ).

Although all these systems have useful proven tools in evaluating risk of death and recurrence, they do present some complexity as many factors need to be assessed. It would therefore be very interesting if there was a unique marker which could be used at the start of follow-up to predict which patients need more aggressive treatment. In this context emerges the role of measuring thyroglobulin (Tg) serum level which has already been demonstrated as a prognosis predictor in DTC ( 9 – 12 ). Its levels, straight after initial treatment, can help determine the presence of persistent or recurrent disease and represent an independent prognosis factor for treatment success ( 11 ).

After TT, Tg seems to reach its lowest level at around three to four weeks ( 2 ), and its levels are influenced by various factors, including the presence of anti-Tg antibodies (TgAb) ( 6 , 13 , 14 ), the remaining quantity of thyroid tissue, the presence of metastatic lesion, time since surgery, and characteristics inherent to the test used for taking measurements ( 2 , 15 ). Also, TSH level at the time of Tg measurement could have an impact on marker concentration ( 2 , 15 ). In fact, although that both basal Tg (during levothyroxine treatment) and stimulated Tg (by high TSH levels; STg) can be used ( 2 ), in about 20% of patients with undetectable basal Tg, the marker is elevated when TSH is higher, which could indicate a worse prognosis ( 16 , 17 ). Thus, STg, obtained both with endogenous TSH and with recombinant TSH use, could be more sensitive than basal Tg ( 17 ).

To measure STg under stimulation with endogenous TSH, it is generally necessary to suspend levothyroxine for three to four weeks, aiming at a significant increase in TSH. Unfortunately, the ideal TSH level to stimulate Tg production by the remaining thyroid/neoplastic cells has not yet been fully established. Concentrations above 30 mU/L seem to be necessary, however, levels between 60 and 90 mU/L would be more reliable for evaluating the marker ( 18 ). It is also difficult to compare different STg measurements and the marker level evolution considering the possibility that TSH concentration may be different in each sample. For example, would the same STg values obtained with TSH levels of 31 or 90 mU/L have the same meaning? In this sense, considering pituitary hormone level, the STg/TSH ratio could have a greater potential for comparison and be a better predictor of therapeutic success than the isolated STg level. The STg/TSH ratio has already proved to be a reliable marker in predicting ^131^I ablative/therapeutic success ( 11 ). In fact, when obtained in the pre-ablative period, this ratio seems valuable in predicting both the ablative success ( 19 ), and the presence of metastases ( 15 ). No references were found on using the STg/TSH ratio to assess long-term case evolution. Thus, this study aimed to compare STg/TSH ratio and first STg level in their ability to predict long-term response to initial therapy.

## SUBJECTS AND METHODS

This study was approved by the Botucatu Medical School Ethics Committee (CAAE no. 83473918.6.0000.5411; Reference no. 2.532.645). This retrospective study compared STg/TSH ratio and first post-thyroidectomy STg level (1^st^ STg) for capacity to predict therapeutic response in DTC patients at final evaluation and during follow-up using classification from the latest ATA guidelines ( 2 ). Data were collected from medical records of DTC patients evaluated in a tertiary hospital.

### Patients

We evaluated 278 DTC patients followed at a specialised out-patient clinic and selected 181 cases (65.1%) according to the following criteria. Inclusion criteria were: cases with anatomopathological DTC diagnosis, submitted to this service's standardised initial treatment between 2001 and 2015 (the period where a standard Tg measuring method was maintained), which at that time consisted of TT followed by ^131^I therapy (DTI); who had Tg, TgAb and TSH results evaluated 1 to 3 months after TT and before DTI; and who had had clinical, laboratory, cervical ultrasound exam follow-up for at least 24 months after initial treatment. Exclusion criteria were: patients who presented positive TgAb; and those with TSH and Tg levels measured from different samples and by different methodologies.

### Variables of interest and evaluated outcomes

The main variables of interest in this study were 1^st^ STg concentrations evaluated between 1 to 3 months post-TT but before DTI, and the ratio between 1^st^ STg and TSH (STg/TSH). Evaluated outcomes were response to treatment at final evaluation and survival time maintained in excellent/indeterminate response. Response to therapy was assessed according to latest ATA therapy response guidelines: excellent, biochemical incomplete, structural incomplete, and indeterminate response ( 2 ). According to this classification, patients were categorised as excellent/indeterminate response or incomplete (biochemical and/or structural) response.

The following patient data were also collected: gender; referred race, white or non-white; age a diagnosis in years; neck dissection performed; anatomopathological diagnosis, according to previous recommendations ( 20 ); staging with regard to risk of death, according to AJCC/TNM 7^th^ edition classification system ( 21 ); and with regard to ATA predicted risk of recurrence (low, intermediate, or high) ( 2 ); result of 1^st^ whole body scan (WBS; considered positive when with cervical or distant uptake after administration of a ^131^I tracing dose), total accumulated therapeutic ^131^I dose; response to therapy 1 year after initial treatment, and follow-up time in months.

### Methods

Treatment and follow-up of DTC patients was performed during the data collection period for this study, as previously reported ( 11 ). Briefly, this included TT, reassessment 1 to 3 months after surgery with STg measured by endogenous TSH (1^st^ STg), WBS, and neck ultrasound (US). Patients subsequently received DTI and were submitted to post-dose WBS about 5 days after treatment. A new evaluation was performed 1 year after DTI with diagnostic WBS, cervical US, and STg and TSH levels. During follow-up, patients were re-evaluated every four to six months with clinical examination and measurements of free thyroxin (FT4), TSH, Tg and TgAb, as well as annual neck ultrasound. If findings suggested persistent or recurrent disease, other imaging tests such as computed tomography, magnetic resonance and positron emission computed tomography (PET-CT) were requested, and if necessary cytohistological exams.

Measurements of FT4, TSH and Tg were performed the Service's Clinical Laboratory using chemiluminescence (DPC, Los Angeles, CA, USA), with the following reference values (RV): 0.80-1.90 ng/dL, 0.40-4.0 µIU/mL and 0.83-68.0 ng/mL, respectively. Analytical Tg sensitivity was 0.2 ng/mL, while functional sensitivity was 0.9 ng/mL (for levels higher than 2 ng/mL). TgAb was measured at the same laboratory by chemiluminescence (Immulite 2000, Siemens, Llanberis, Gwynedd, United Kingdom), with manufacturer RV of ≤ 40 UI/mL, above which it was considered positive.

### Statistical analysis

Collected data were tabulated in Excel^®^ (Microsoft Corporation, USA) and submitted to statistical analysis using SAS v9.4 software. Qualitative variables were expressed as frequencies and percentages and evaluated using the Chi-squared and Fisher Exact tests. Quantitative variables were expressed and means and standard deviations and evaluated by the Student t test. In general, a 5% (p < 0.05) significance level was adopted. However, variables with p ≤ 0.15 were submitted to multivariate logistic regression with incomplete (biochemical/structural) response to initial therapy as the response variable. This higher than usual level of significance (0.15) was used to minimize the risk of neglecting important variables for the outcome. ROC (receiver-operating characteristic) curves were constructed for the 1^st^ STg and STg/TSH ratio to establish cutoffs which could predict incomplete therapeutic (biochemical/structural) response, with respective area under the curve (AUC), sensitivity, specificity, positive predictive value (PPV), negative predictive value (NPV) and accuracy. Considering these cutoffs, Kaplan-Meyer curves were also constructed to evaluate survival time in excellent/indeterminate response.

## RESULTS

Clinical, histopathological, therapeutic, and evolution data of the studied cases can be found in Table 1 . The majority (n = 158; 87.3%) were female and declared race white (n = 169; 93.4%). Seventy-five patients (41.4%) were submitted to lymph node dissection. The most frequent histological type was CP (n = 163; 90.1%), with 117 cases (64.6%) having presented TNM Stage I and 99 (54.7%) displaying low risk of recurrence. The majority of cases (n = 170; 94.4%) presented positive at 1^st^ WBS, and of these most had only had neck uptake (n = 163; 95.9%). Accumulated mean DTI [±standard deviation (SD)] was 158.77 (±56.92) mCi. Mean (±SD) values of 1^st^ STg and STg/TSH ratio were 35.11 (±245.29) ng/mL and 0.47 (±3.28), respectively. One hundred and sixteen patients (64.1%) presented an excellent response 1 year after initial treatment, while 120 (66.3%) presented this same response at final evaluation, with a mean (±SD) follow-up time of 87.24 (±44.94) months (median = 76, minimum = 24, maximum = 188 months). Twenty-eight cases (15.5%) presented incomplete (biochemical or structural) response at some time during follow-up.

**Table 1:** Clinical, histopathological, therapeutic, and evolutionary data of the studied population

Data			
Female, n (%) a			158 (87.3)
White color reported, n (%) a			169 (93.4)
Age (years) b			48.45 ± 14.19
Lymph node dissection, n (%) a			75 (41.4)
Type of cancer			
	Papillary carcinoma, n (%) a		163 (90.1)
		Classical variant, n (%) a	101 (62.0)
		Follicular variant, n (%) a	51 (31.3)
		Oncocytic variant, n (%) a	6 (3.7)
		Sclerosing variant, n (%) a	2 (1.2)
		Solid variant, n (%) a	3 (1.8)
	Follicular carcinoma, n (%) a		18 (9.9)
Staging (TNM)			
		I, n (%) a	117 (64.6)
		II, n (%) a	25 (13.8)
		III, n (%) a	24 (13.3)
		IV, n (%) a	15 (8.3)
Risk of recurrence			
		Low, n (%) a	99 (54.7)
		Intermediate, n (%) a	52 (28.8)
		High, n (%) a	30 (16.6)
Positive 1st WBS, n (%) a			170 (94.4)
		Cervical uptake, n (%) a	163 (95.9)
		Cervical and distant uptake, n (%) a	7 (4.1)
RIT total dose (mCi) b			158.77 ± 56.92
1^st^ STg (ng/mL) b			35.11 ± 245.29
STg/TSH ratio b			0.47 ± 3.28
Therapeutic response in one year			
		Excellent, n (%) a	116 (64.1)
		Indeterminate, n (%) a	50 (27.6)
		Incomplete biochemistry, n (%) a	3 (1.7)
		Incomplete structural, n (%) a	12 (6.6)
Incomplete response at some point, n (%) a			28 (15.5)
Therapeutic response in the last evaluation			
		Excellent, n (%) a	120 (66.3)
		Indeterminate, n (%) a	48 (26.5)
		Incomplete biochemistry, n (%) a	3 (1.7)
		Incomplete structural, n (%) a	10 (5.5)
Follow-up time (months) b			87.24 ± 44.96

1^st^ STg: first stimulated thyroglobulin; mCi: milicuries; n: number; %: percentage; RTI: radioactive iodine ablation/therapy; TNM: American Joint Commission on Cancer (AJCC) tumor staging system; TSH: thyrotropin; WBS: whole-body scan.

a Frequencies and percentages for categorical variables.

b Mean ± standard deviation.

Cases with incomplete (biochemical or structural) response at final evaluation were compared with those with excellent/indeterminate response ( Table 2 ), with the former group displaying higher serum concentrations of 1^st^ STg [225.13 ± 585.26 ng/mL *versus* ( *vs* ) 20.4 ± 192.9 ng/mL; p < 0.001] and STg/TSH ratio (3.01 ± 7.8 *vs* 0.27 ± 2.58; p < 0.001). The patients with incomplete response also presented a higher percentage of cases of high risk of recurrence (38.5% *vs* 14.9%; p = 0.078) and received higher accumulated doses of ^131^I (383.8 ± 286.6 mCi *vs* 168.02 ± 86.51 mCi; p < 0.0001). The groups did not differ in the other evaluated parameters.

**Table 2:** Comparison between patients with excellent/indeterminate and incomplete response (biochemical and/or structural) in the last evaluation, regarding clinical, laboratory, histopathological, and therapeutic data

Data		Response		P
		Excellent/Indeterminate n = 168 (92.8%)	Incomplete (Biochemical/Structural) n = 13 (7.2%)	
Female, n (%) a		146 (86.9)	12 (92.3)	0.573
Age (years) b		48.24 ± 13.75	51.15 ± 19.48	0.476
Lymph node dissection, n (%) a		68 (40.5)	7 (53.8)	0.345
Type of cancer				0.100
	Papillary carcinoma, n (%) a	153 (91.1)	10 (76.9)	
	Follicular carcinoma, n (%) a	15 (8.9)	3 (23.1)	
Staging (TNM)				0.230
	I, n (%) a	110 (65.5)	7 (53.8)	
	II, n (%) a	23 (13.7)	2 (15.4)	
	III, n (%) a	23 (13.7)	1 (7.7)	
	IV, n (%) a	12 (7.1)	3 (23.1)	
Risk of recurrence				**0.078**
	Low, n (%) a	93 (55.4)	6 (46.1)	
	Intermediate, n (%) a	50 (29.8)	2 ( 15 , 4 )	
	High, n (%) a	25 (14.9)	5 (38.5)	
Positive 1st WBS, n (%) a		157 (94)	13 (100)	1.00
RIT total dose (mCi) b		168.02 ± 86.51	383.8 ± 286.6	**<0.0001**
1^st^ STg (ng/mL) b		20.4 ± 192.9	225.13 ± 585.26	**<0.001**
STg/TSH ratio b		0.27 ± 2.58	3.01 ± 7.8	**<0.001**
Follow-up time (months) b		88.16 ± 45.36	75.38 ± 39.06	0.325

1 1^st^ STg: first stimulated thyroglobulin; mCi: milicuries; n: number; %: percentage; RTI: radioactive iodine ablation/therapy; TNM: American Joint Commission on Cancer (AJCC) tumor staging system; TSH: thyrotropin; WBS: whole-body scan.

a Frequencies and percentages; Chi-square and Fisher's exact tests.

b Mean ± standard deviation; Student's t-test. Statistical significance: p < 0,05.

In the multivariate analysis, performed with variables where p ≤ 0.15, the type of cancer [for FC; odds ratio (OR) = 5.552; confidence interval (CI): 1.082-28.5; p = 0.04] and accumulated DTI (OR = 1.008; CI: 1.003-1.013; p = 0.001) continued as significant.

**Figure 1:**
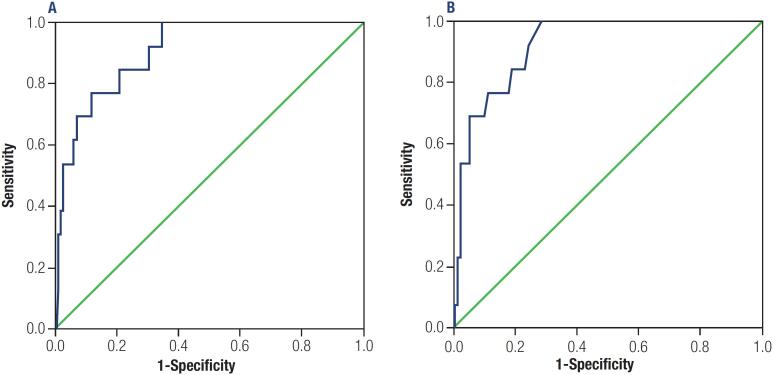
Receiver-operating characteristic (ROC) curves of the first stimulated thyroglobulin [A: cutoff = 5.0 ng/mL ng/mL (area under the curve: 0.907; p < 0.001)] and regarding first stimulated thyroglobulin/thyroid-stimulating hormone ratio [B: cutoff = 0.085 (area under the curve: 0.920; p < 0.001)] as predictors of incomplete response (biochemical and/or structural) in the last evaluation.

ROC curves were constructed to establish cutoff points for 1^st^ STg and STg/TSH values that could predict a higher risk of the patient presenting incomplete response at last evaluation ( Figure 1 ). The cutoff obtained for 1^st^ STg was 5 ng/mL, with AUC of 0.907 (p < 0.001; CI 95%: 0.84-0.97), 76.7% sensitivity, 79.2% specificity, 22.2% VPP, 97.8% VPN and 79% accuracy. For the STg/TSH ratio cutoff was 0.085, with AUC of 0.920 (p < 0.001; CI 95%: 0.87-0.97), 76.9% sensitivity, 82.6% specificity, 25.6% VPP, 97.8% VPN and 82.2% accuracy.

**Figure 2:**
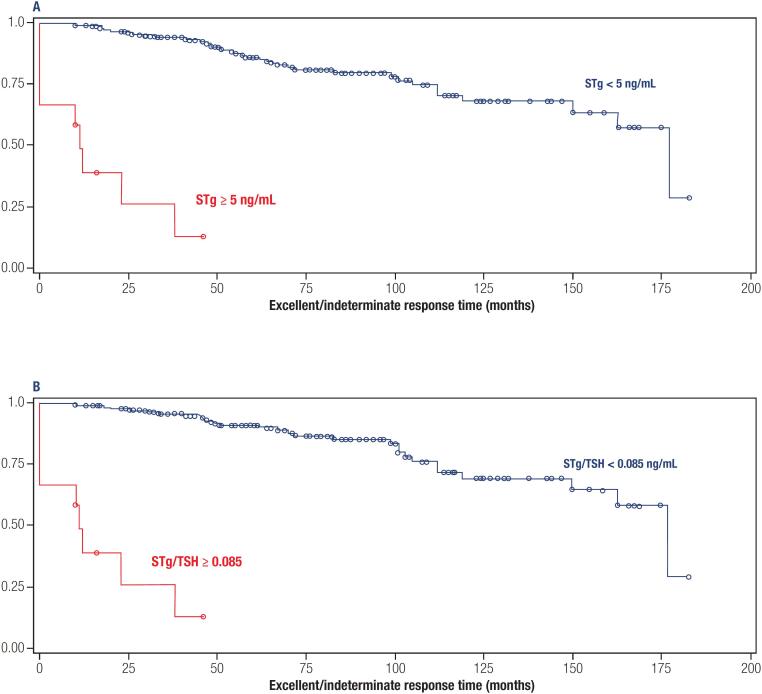
Kaplan-Meyer curves to assess patient survival time in excellent/indeterminate response, using the cutoff point established for the first stimulated thyroglobulin – STg as variable (A: mean survival with STg ≥ 5 ng/mL = 15.9 months and with STg < 5 ng/mL = 140.4 months; Log Rank < 0.001) and for the first stimulated thyroglobulin/thyrotrophin – STg/TSH ratio (B: mean survival with STg/TSH ≥ 0.085 = 15.9 months and with STg/TSH < 0.085 = 144.6 months; Log Rank < 0.001) in the last evaluation as predictors of outcome.

The cases with 1^st^ TgS ≥ 5 ng/mL showed survival maintained in an excellent/indeterminate response for an average time of 15.9 months, whereas in those with 1^st^ Tg < 5 ng/mL, this time was 140.4 months. In patients with STg/TSH ratio ≥ 0.085, this time was 15.9 months against 144.6 months in those with STg/TSH ratio < 0.085 ( Figure 2 ).

## DISCUSSION

In this study we demonstrated that both 1^st^ STg and the STg/TSH ratio, evaluated 1-3 months after TT and before 1^st^ DTI, showed an association with long-term prognosis for DTC. Tg, and particularly STg, is already recognised as a prognosis and remission marker for the disease ( 17 ). However, as it is influenced by TSH level, it is interesting to evaluate the STg/TSH ratio, which has been associated with metastases detection and DTI effectiveness ( 11 , 15 , 19 ), but not until the completion of this study, had its potential in long-term prognosis been assessed.

In this study, after a mean follow-up time of 87 months, patients with excellent/indeterminate response at final consultation presented 1^st^ STg lower than cases with incomplete (biochemical/structural) response. Concentrations equal or greater than 5 ng/mL were predictors of incomplete response at final consultation with elevated sensitivity, specificity, VPN, and accuracy (76.7%, 79.2%, 97.8% and 79%, respectively). Only VPP was lower, possibly influenced by the relatively small number of patients with incomplete/indeterminate response. Evaluating intermediate-risk patients, Faro and cols. observed a higher cutoff point for the marker, with STg levels> 10 ng/mL having been associated with an incomplete response ( 22 ). Perhaps the reason for the difference observed could be the time to assess the outcome, which in that study was 12 to 18 months ( 22 ). Interestingly, the 1^st^ STg cutoff value observed in this study was very close to that seen in an earlier study by the same service (4.41 ng/mL), and which proved to be a predictor of DTI ablative success ( 11 ). STg was also a predictor of maintenance time in response to excellent/indeterminant response during follow-up, where cases with STg < 5 ng/mL having presented a time around 8.8 times longer than those where the marker was above this level. In fact, 1^st^ STg is recognised as a prognosis marker for DTC patients where levels below 1 ng/mL have been associated with absence of structural disease with prediction rates over 90% ( 7 ). However, the capacity of 1^st^ STg in predicting long-term disease absence has been variable, with VPNs between 69.8% and 94.2% having been reported ( 7 , 23 ). As for the association between 1^st^ STg and maintenance time in excellent response, this has generally not been a focal point in different studies thus making comparison difficult.

As already cited, despite its excellent performance, STg can be influenced by TSH level. In fact, although patients with excellent or incomplete response do not seem to significantly differ in terms of STg levels, when these are obtained with TSH values between 30-60 mU/L, higher concentrations of the pituitary hormone can cause more significant elevations in the marker, providing differentiation between these groups ( 18 ). It is true that, with the advent of recombinant TSH, this discussion could seem to be without reason. However, this is not yet widely available to the general population, and significant numbers of patients, especially those monitored in the public health service, still depend on endogenous TSH elevation for performing STg measurements. A limiting factor in this approach is that the achievement of much higher endogenous TSH levels generally implies the absence of levothyroxine treatment for long periods, which in turn can lead to a higher incidence of adverse effects and impact quality of life ( 24 – 26 ). In this context, correcting the marker by TSH concentration at time of evaluation, which occurs when using the STg/TSH ratio, seems more appropriate. In this study, we observed that patients with incomplete response at final evaluation had presented much higher values of this ratio soon after initial treatment. Values equal to or greater than 0.085 have even been shown as predictors of incomplete response, with high sensitivity, specificity, VPN and accuracy (76.9%, 82.6%, 97.8% and 82.2%, respectively). As with STg, only PPV was low. This cutoff value was very close to that seen in a previous study at the same service (0.093) which proved to be a predictor of DTI ablative success ( 11 ). However, it was much lower than the value reported by other authors who also evaluated ablative success ( 19 ). In our study, this ratio was also a predictor of maintenance time in excellent/indeterminate response during follow-up, with cases presenting an STg/TSH ratio < 0.085 having an around 9 times longer maintenance time than those where the ratio value was higher. No studies were found evaluating STg/TSH ratio regarding maintenance time in a given response.

This study has limitations, such as its retrospective character and the not very large sample number, particularly in relation to incomplete response cases, which could have contributed the findings obtained in analysis of association not being maintained in multivariate analysis, for instance reduced VPP seen for STg and STG/TSH ratio cutoffs. However, this study has merit in presenting the STg/TSH ratio as an additional tool for predicting long-term prognosis, considering response outcome to initial therapy in the light of the most recent recommendations for monitoring DTC. This ratio could be particularly useful in situations where the increase obtained in endogenous TSH has not been so significant.

In conclusion, our results indicate that the STg/TSH ratio has a similar performance to the 1^st^ STg in predicting long-term response to initial therapy. Thus, these are both useful markers in evaluating prognosis for DTC patients. More studies with larger samples are necessary to investigate the real role of the STg/TSH ratio in following up these patients.
